# Protocol for a systematic review and economic evaluation of the clinical and cost-effectiveness of non-hospital-based non-invasive ventilation (NIV) in patients with stable end-stage COPD with hypercapnic respiratory failure

**DOI:** 10.1186/2046-4053-3-32

**Published:** 2014-03-27

**Authors:** Chirag Dave, Alice Turner, Janine Dretzke, Sue Bayliss, Deirdre O’Brien, Sue Jowett, David Moore

**Affiliations:** 1 Heart of England Trust, Heartlands Hospital, Bordesley Green East, Birmingham B9 5SS, UK; 2 Centre for Translational Inflammation Research, School of Clinical and Experimental, Medicine, University of Birmingham, Edgbaston, Birmingham B15 2TT, UK; 3 Department of Public Health, Epidemiology and Biostatistics, School of Health and Population Sciences, University of Birmingham, Edgbaston, Birmingham B15 2TT, UK; 4 Health Economics, School of Health and Population Sciences, University of Birmingham, Edgbaston, Birmingham B15 2TT, UK

**Keywords:** COPD, Non-hospital-based non-invasive ventilation, Hypercapnia, Clinical effectiveness, Cost-effectiveness, Systematic review, Meta-analysis

## Abstract

**Background:**

Chronic obstructive pulmonary disease (COPD) remains a significant public health burden. Non-invasive ventilation (NIV) is a method of supported breathing used as standard care for acutely unwell patients in hospital with COPD, but there is uncertainty around the potential benefits of using NIV in the treatment of stable patients in a non-hospital setting. This is a protocol for systematic reviews of the clinical and cost-effectiveness of NIV in this context, being undertaken in support of a model based economic evaluation.

**Methods/Design:**

Standard systematic review methods aimed at minimising bias will be employed for study identification, selection and data extraction for both the clinical and economic systematic reviews. Bibliographic databases (for example MEDLINE, EMBASE) and ongoing trials registers will be searched from 1980 onwards. The search strategy will combine terms for the population with those for the intervention. Studies will be selected for review if the population includes adult patients with COPD and hypercapnic respiratory failure, however defined. Systematic reviews, randomised controlled trials and observational studies (with n >1) will be included, and quality assessment will be tailored to the different study designs. The primary outcome measures of interest are survival, quality of life, and healthcare utilisations (hospitalisation and Accident and Emergency attendances). Meta-analyses will be undertaken where clinical and methodological homogeneity exists, supported by predefined subgroup analyses where appropriate. A systematic review of the evidence on the cost-effectiveness of non-hospital NIV will be completed, and a model-based cost-utility analysis undertaken to determine the cost-effectiveness of non-hospital-based NIV compared with standard care.

**Discussion:**

These reviews will attempt to clarify the clinical effectiveness of non-hospital NIV in COPD patients as well as the cost-effectiveness. The findings may indicate whether NIV in a non-hospital setting should be considered more routinely in this patient group, and what the likely cost implications will be.

**PROSPERO registration:**

2012:CRD42012003286.

## Background

Chronic obstructive pulmonary disease (COPD) is a progressive lung disease, characterised by non-reversible airflow obstruction, mostly affecting middle-aged or elderly people who have smoked [1]. It is projected that COPD will be the third leading cause of death worldwide by 2020 [2]. Currently, treatment is mainly symptomatic and aims to slow down disease progression. The main evidence-based treatments are inhaled agents, such as bronchodilators [1], and pulmonary rehabilitation [3]. Patients will have periods of no change in symptoms, and this is often considered a stable state.

As the disease progresses the lungs are unable to perform two of their basic functions: to get oxygen to the bloodstream, and to eliminate carbon dioxide. Hypoxia is the presence of low oxygen levels and long-term oxygen therapy (LTOT) is considered in selected patients [1]. Hypercapnia describes a high carbon dioxide level, the presence of which in a stable patient is a poor prognostic sign [4]. When the respiratory system fails like this, a patient could be considered to be in the end stage of their disease. Classically, end-stage COPD would be defined as those patients in the terminal stage of their disease, likely to die within months – a situation that is not always clear [5]. Alternatively, it might be defined as those who have developed chronic respiratory failure and remain symptomatic on maximal therapy, with no hope of cure.

Non-invasive ventilation (NIV) is a method of providing ventilatory support via a mask, without the placement of an endotracheal tube [6]. It is commonly used in the hospital setting as standard care for acutely unwell patients with COPD [7]. As the technology has improved and NIV devices have become less cumbersome, an increasing number of patients have been able to use the devices outside of hospital [8]. This includes patients with chronic respiratory failure due to kyphoscoliosis and neuromuscular disorders, for whom domiciliary NIV is an established treatment. Some centres also advocate NIV in a domiciliary setting for stable COPD patients with chronic hypercapnic respiratory failure. Physiological studies have shown improvements in lung function and carbon dioxide levels thought to be due to eliminating nocturnal hypoventilation [9]. Some evidence suggests that it prevents episodes of recurrent acute hypercapnic respiratory failure and hospital admissions [10].

However based on a recent systematic review of randomised controlled trials (RCTs), evidence on the benefit of home NIV in this patient group remains inconclusive, particularly regarding long-term outcomes [11]. Variations between the included RCTs in terms of study methods and physiological or clinical outcomes measured, together with a lack of adjustment for clinical variables (such as oxygen use or prior acute NIV use) have limited the conclusions that can be drawn. Furthermore the RCTs, for the most part, appear to have insufficiently long follow-up periods to capture outcomes relating to survival, long-term quality of life (QoL), exacerbations over the long term, adverse events or adherence rates, all of which are important in considering cost-effectiveness. Scoping searches suggest that observational studies may have included larger sample sizes, longer follow-up periods, additional outcome measures and less restrictive inclusion criteria than randomised trials and thus may have wider applicability.

Cost or cost-effectiveness evidence of non-hospital NIV in patients with stable end-stage COPD is sparse. There are no systematic reviews of the cost-effectiveness of non-hospital NIV. Some economic evaluations have been undertaken either in parallel with clinical trials or through audits of patient records [10,12]. However, they appear not to be based on systematic review of clinical effectiveness, therefore, a robust evaluation of the literature and development of a new economic model is warranted.

## Study design

### Aims and objectives

The aim is to undertake a systematic review of the evidence on the clinical and cost-effectiveness of home or wider non-hospital-based NIV in patients with stable, end-stage COPD (however defined).

### Clinical effectiveness

● A review of the existing systematic reviews of RCTs on the clinical effectiveness of non-hospital-based NIV.

● A systematic review of RCTs on the clinical effectiveness of non-hospital-based NIV.

● A systematic review of observational studies on the clinical effectiveness of non-hospital-based NIV.

### Cost-effectiveness

● A systematic review of the evidence on the cost-effectiveness of non-hospital-based NIV.

● A model-based cost-utility analysis to determine the cost-effectiveness of non-hospital-based NIV.

### Methods

Standard systematic review methodology aimed at minimising bias will be employed. The National Institute of Health Research (NIHR), Health Technology Assessment (HTA) programme, has ethically approved this review. This protocol is registered with PROSPERO (2012:CRD42012003286).

### Searches

The following sources will be searched for both clinical and cost-effectiveness reviews:

● Bibliographic databases - MEDLINE, MEDLINE In Process and EMBASE via Ovid, CINAHL via EBSCO, Cochrane Library (CDSR, DARE, HTA, NHS EED and CENTRAL databases), Science Citation Index (ISI)

● Current controlled trials *meta*Register, ISRCTN database, UKCRN, WHO ICTRP Portal and ClinicalTrials.gov for ongoing studies

● Specialist abstract and conference proceeding resources (British Library’s ZETOC and ISI Proceedings)

● Consultation with experts in the project research team field

● Checking of citation lists of included studies and relevant systematic reviews

● Contact with study authors and researchers of ongoing trials

Given that the main co-intervention/comparator, LTOT, has been in routine use since the 1980s, searches will be run from this time point. The strategies will include a combination of text words and index terms relating to NIV and COPD as appropriate. There will be no language restrictions applied to the searches. Study design filters will not be used. Search results will be entered into electronic databases (Reference Manager v11 Thomson ISI ResearchSoft) to facilitate record keeping, duplicate removal, study selection and document writing.

Titles (and abstracts where available) of articles identified by the searches will be screened by two reviewers, independently, for relevance to the review question using pre-specified screening criteria. This process will be aimed at removing non-relevant studies. Hard copies of relevant articles will be acquired and assessed against the inclusion criteria (see below) specific to the review being undertaken by two reviewers independently. Discrepancies between reviewers will be resolved by discussion or by referring to a third reviewer. Where necessary, translation (full/part) of non-English language articles will be undertaken to facilitate this process and subsequent reviewing; the review team has access to a wide range of translators. The study selection process will be illustrated using a PRISMA flow diagram [13]. Reference management software will be used to record reviewer decisions, including reasons for exclusion.

## Clinical effectiveness

### Selection criteria

A sample search strategy, for the clinical effectiveness review, for MEDLINE is provided in Appendix 1.

#### Study design

Systematic reviews will be included in order to obtain an overview of the existing evidence base. RCTs will be included with no restrictions on the type of RCT (for example parallel, cross-over). In view of the steady deterioration in the health of patients with severe COPD, however, it seems unlikely that cross-over studies will be a suitable method to make unbiased comparisons of the long-term efficacy of home NIV. All observational evidence will be obtained, whether controlled or uncontrolled, in order to gain an overview of existing observational evidence. Uncontrolled observational studies will be used where primary outcomes are not reported in the control studies, or, where uncontrolled studies have longer follow-up for these outcomes.

#### Patient group

The patient group will be adult patients with stable end-stage COPD plus chronic hypercapnic respiratory failure, who have required assisted ventilation (whether invasive or non-invasive) during an exacerbation or who are hypercapnic or acidotic on LTOT, providing they do not require treatment in hospital. The criteria for specifying the population are broad, to include any adult patients with COPD and hypercapnic respiratory failure, however defined, and inclusion will not be restricted by disease severity. Where a study contains a mixed population, the study will be included, but inclusion into analysis will only be possible where data are available separately for the relevant population or if the majority of the population are relevant. In the latter case, such a study’s effect on summary data will be investigated by sensitivity analyses.

#### Technology

Studies of any form of NIV will be included, whether continuous or intermediate, added to (any form of) standard care. Previous research has shown that patients in acute settings show improvement after four hours [14], however, study inclusion will not be restricted by length of daily use.

#### Comparators/control

For controlled studies

a) Any form of standard care with no NIV; it is noted that both the setting and the nature of standard care in the absence of treatment with NIV may be different to that of treatment with NIV; such differences will not affect inclusion/exclusion decisions, but will be noted and commented upon and considered in the analyses.

b) Studies comparing alternative methods of NIV will also be included. The main difference is likely to be whether NIV is set at pressure or volume controlled. Other differences include mask type and number of hours of use per day.

#### Setting

Home or wider non-hospital setting, operationally this equates to a non-hospital environment.

#### Outcome

Studies will be included if they contain any outcomes related to patient wellbeing, healthcare service utilisation and/or patient carers. Based primarily on the need to inform an economic model, we consider the main outcomes for the review to be:

Primary outcomes

● survival

● QoL with validated questionnaires for patient and carer (for example EQ5D, SF-36, St George’s Respiratory Questionnaire)

● exacerbations (and requirements for associated medication)

● hospitalisations

● Accident and Emergency admissions

Secondary outcomes

● other healthcare resource use (for example primary care, training)

● lung function (for example, forced expiratory volume in one second (FEV_1_), forced vital capacity (FVC))

● blood gases (for example, partial pressure of carbon dioxide in arterial blood (PaCO_2_))

● dyspnoea

● (serious) adverse events (for example, barotrauma, pneumonia, nasal skin lesions)

● other patient- or carer-related outcomes such as quality of sleep, activities of daily living and acceptability

● adherence/compliance rates

#### Data extraction

Data extraction will be conducted independently by two reviewers using a standardised extraction form. Disagreements will be resolved through discussion or referral to a third reviewer. For each study, the data required on (but not limited to) the following will be sought:

Study characteristics

● country of origin

● study design

● setting

● sample size

● length of follow-up

Population

● patient inclusion and exclusion criteria

● patient characteristics

● proportion on LTOT

Intervention/comparator

● NIV versus standard care or NIV versus alternative NIV

● details of standard care (including additional oxygen therapy)

● type of NIV equipment; pressure or volume controlled

● length of nocturnal/daytime NIV

● patient training/education provided

● run-in period (duration/setting)

● patient adherence/compliance (how assessed/reported)

Results

● completeness of follow-up

● outcome measures

● statistical methods employed

● findings

● effect sizes and associated uncertainty

### Quality assessment

Data will be extracted to allow quality assessment of the included studies. Study quality will be assessed using tools specific to a given study design. For systematic reviews, the AMSTAR checklist will be used [15]. The risk of bias tool from the *Cochrane Handbook* will be used for RCTs [16]. Should cross-over trials be included, then additional areas of risk of bias will need to be assessed. These relate to: (i) whether the cross-over design is suitable; (ii) whether there is a carry-over effect; (iii) whether only first-period data are available; (iv) appropriate statistical analysis; and (v) comparability of results with those from parallel-group trials [17].

For observational studies, the guidelines outlined in Chapter 13 of the *Cochrane Handbook* will be followed [18]. For controlled observational studies the domains in the risk of bias tool for RCTs can be used as a minimum assessment (accepting that the studies are not randomised). The most relevant criteria for assessment in this area are likely to relate to how the groups were selected, differences in patient characteristics, loss to follow-up and biases and confounding in outcome assessment.

For uncontrolled observational studies a tailored assessment tool will be developed for this review based on existing tools (such as the Downs and Black instrument [19] or the Newcastle Ottawa scale [20]). Criteria such as a clear description of population characteristics, use of validated outcome measures and reporting of length and loss to follow-up are likely to be relevant.

In addition to methodological criteria listed above, the GRADE framework [21] will be used to consider inconsistency between studies, precision of results, likelihood of publication bias and applicability of results to population(s) of interest.

### Analysis

Narrative synthesis of evidence will be undertaken for all included studies. Results are likely to be presented using a number of different outcome statistics, for example, mean difference, relative risk, hazard ratio and so on. This may be the case even for the same outcome, for example, admissions to hospital could be reported as mean number of admissions per patient or total number of admissions. Survival could be reported as time-to-event data or as relative risk. Time points of reporting are also likely to vary across studies. The relevance of short-term outcome assessment is often related to underlying population risk for the outcome in question. For example, patients discharged from hospital after intensive emergency treatment for an exacerbation of their COPD are at higher risk of a recurrent exacerbation in the immediate aftermath (for example three months) than patients who have been stable without a severe exacerbation for many months. Where appropriate, meta-analytic methods will be employed to combine data reported by the same outcome statistic across the same, or very similar time points; summary statistics will most likely be pooled relative risk for dichotomous outcomes, pooled mean difference for continuous outcomes or pooled hazard ratios. This may involve conversion of different statistics into a single, consistent measure, where appropriate assumptions are met, for example by using the method of Parmar to obtain hazard ratios from dichotomous data [22]. Standardised mean differences will be considered if the same outcome is measured using different assessment tools. Final choice of summary statistic and method of meta-analysis will be guided by the considerations outlined in Chapter 9 of the *Cochrane Handbook*[23]. Assessment of clinical and methodological heterogeneity will be used to determine whether a fixed or random-effects model is the most appropriate, rather than relying on the tests of heterogeneity from a fixed-effect model to make such a decision [24]. The I^2^ statistic (which gives the percentage of the total variability in the data due to between-study heterogeneity) and the tau-squared statistic (which gives an estimate of the between-study variance) will be reported where appropriate. Evidence from RCTs and observational studies will not be quantitatively combined, but presented separately.

Consideration may need to be given on whether to incorporate cross-over trials into any meta-analyses. Should cross-over trials be included in a meta-analysis then separate analyses for parallel and cross-over studies will also be presented [17].

For each meta-analysis containing 10 or more studies, the likelihood of publication bias will be investigated through the construction of funnel plots and appropriate statistical tests for small-study effects (such as the Peters Test [25]); that is, the tendency for smaller studies to provide more positive findings. It is well recognised that, especially where heterogeneity exists, publication bias may be one of a number of reasons for any small-study effects identified. The restriction of 10 studies is due to the low power of identifying small-study effects with few studies [16]. Where studies have reported time-to-event analyses, meta-analysis using the extracted hazard ratios and their variances will be undertaken, if possible.

The potential for indirect comparisons/multiple treatment comparisons will be explored, for example if there are RCTs comparing different types of NIV interventions respectively, but with a common comparator (for example, NIV_1_ versus standard care and NIV_2_ versus standard care). A number of key assumptions would have to be met, including that of homogeneity and exchangeability of participants between trials [26]. The similarity of trial and population characteristics within and between trials will therefore be assessed qualitatively and statistically; however, if the trials are small there will be limited power to assess the statistical inconsistency of any direct and indirect comparisons. If such comparisons are deemed possible, a Bayesian approach, to take into account parameter uncertainty and allow for probability statements and ranking of treatment modalities, will be used. It is likely that vague priors will need to be used and sensitivity to variation in these will be investigated.

### Subgroup analysis

As NIV is becoming easier and cheaper to use in practice, knowledge about how and when to use it, as well as in whom it is most effective, becomes of key clinical and budgetary significance. To aid this and to further assist economic model parameterisation (see below) a priori data analysis of relevant subgroups will be undertaken where deemed appropriate. Where data allows, such analysis could include grouping by clinically perceived effect modifiers such as type of ventilation (for example pressures), patient interface (face/nasal mask), number of hours of use per day of NIV (where there are clear differences in trial protocols), patients on/not on LTOT and severity of disease (including frequent versus non-frequent exacerbators). Severity of disease may not always be well described, but where possible populations will be identified according to, for example, the GOLD criteria (2011) [4] and patients classified as ‘very severe’ may be compared to patients of other grades of severity (‘mild’, ‘moderate’, ‘severe’). Sensitivity analysis may be undertaken where, for example, studies contained a mixed population of those relevant and not relevant to the project. We are unlikely to assess the robustness of any meta-analysis conclusions to varying study quality, unless a clear difference in methodological quality is identified between groups of included studies.

## Cost-effectiveness

### Searches

Strategies will include terms relating to cost, cost-effectiveness and QoL.

### Study selection

Inclusion criteria for population, intervention and comparator will be the same as for the clinical effectiveness systematic review. Inclusion criteria for study design will target cost-analysis, cost-effectiveness, cost-utility and cost-benefit studies and decision model-based analyses. Outcomes will be QoL, costs and incremental cost-effectiveness ratios.

### Quality assessment

Quality assessment will be appropriate to the study design, for example, the Drummond checklist [27] for economic evaluations and the Philips checklist [28] for model-based analyses.

### Analysis

Each included study will be narratively reviewed and similarities and differences between studies described and tabulated. A prior assumption is that few economic studies exist, therefore, the primary aim is to confirm or refute this assumption and also to identify additional parameters for the cost part of the economic evaluation not provided by the clinical effectiveness review.

### Economic evaluation

An economic evaluation will be carried out from a National Health Service (NHS) and Personal Social Services (PSS) perspective, to take into account both healthcare and social care costs of COPD. If data on the impact of stable end-stage COPD on carers is available from the literature, then a wider societal perspective will be considered in a sensitivity analysis, taking into account costs and outcomes associated with informal care. The model will determine the cost-effectiveness of NIV in a non-hospital setting, in patients with stable end-stage COPD, compared with standard care.

The economic evaluation will involve developing a Markov-type, state transition model. This is the most appropriate model type for this decision problem as the model can represent a chronic disease such as COPD where patients change health states over time. It is anticipated the model will have a short time cycle (for example a month) to account for exacerbations resulting in Accident and Emergency attendance and hospital admission, which will in turn impact on healthcare costs, QoL and survival. The time horizon of the model will be patient lifetime, which, due to the age of the patients and the high level of mortality, is unlikely, for most patients, to extend beyond five years. Findings from the systematic reviews of clinical and cost-effectiveness will be used to inform the economic evaluation and populate the model.

Additional targeted searches may be performed on an ad hoc basis to obtain information to populate model parameters. Modelling studies that consider NIV treatment in other patient populations (for example muscular dystrophy) may be consulted to identity relevant cost data.

Standard sources of unit costs will be utilised, with additional costs obtained from healthcare providers when no relevant published data can be found. QoL data will be determined from previously published studies. In the absence of QoL information from utility-based measures, data from disease-specific tools such as the St George’s Respiratory Questionnaire will also be sought and a mapping algorithm applied to predict utility scores [29].

For each important model parameter, we will determine a point estimate with a range of possible values (for example confidence intervals) and construct a probability distribution around that point estimate. Base case evaluation will be based on the most likely estimates. Discounting will be applied at the standard rate of 3.5% to both costs and outcomes. Univariate and probabilistic sensitivity analyses will be conducted to deal with uncertainty in model parameters and quantify the overall decision uncertainty. Based on findings from the effectiveness review and estimated costs, the economic evaluation may consider alternative patient subgroups and additional scenarios where appropriate. A value of information (VoI) analysis may be considered, if deemed relevant, in order to explore whether further research is required and to support recommendations for the research agenda in this clinical area.

## Discussion

COPD continues to be a massive health burden worldwide. COPD patients admitted to hospital with an exacerbation are reaping the benefits from improvements in standard medical treatment, including the use of in-hospital NIV. Physiological reasons exist to why the long-term use of NIV outside the hospital setting may prevent hospital admissions for acute exacerbations. There is much variability in the application of non-hospital NIV for COPD and the hope is to identify whether there is an actual benefit to patients and at what cost. NIV is becoming easier and cheaper to use in practice, and knowledge about in whom, how and when to use it is of key clinical and budgetary significance.

## Appendix 1. Sample search strategy for MEDLINE

Database: MEDLINE (Ovid) 1948 to June Week 2 2011

1. chronic obstructive pulmonary disease.mp. or exp Pulmonary Disease, Chronic Obstructive/

2. copd.mp.

3. chronic obstructive lung disease.ti,ab..

4. chronic obstructive airway disease.ti,ab.

5. chronic respiratory disorder$.ti,ab.

6. smoking-related lung disease$.ti,ab.

7. Pulmonary Emphysema/

8. exp Bronchitis/

9. emphysema.mp.

10. or/1-9

11. noninvasive ventilation.ti,ab.

12. non-invasive ventilation.ti,ab.

13. exp positive-pressure respiration/or intermittent positive-pressure ventilation/

14. npvv.ti,ab..

15. cpap.ti,ab.

16. bipap.ti,ab.

17. bi-level ventilation.ti,ab.

18. niv.ti,ab.

19. nippv.ti,ab.

20. positive pressure ventilation.ti,ab.

21. (non-invasive adj2 ventilation).ti,ab.

22. or/11-21

23. 10 and 22

## Abbreviations

COPD: chronic obstructive pulmonary disease; FEV1: forced expiratory volume in one second; FVC: forced vital capacity; LTOT: long-term oxygen therapy; NIV: non-invasive ventilation; PaCO2: partial pressure of carbon dioxide in arterial blood; QoL: quality of life; RCT: randomised controlled trial; VoI: value of information.

## Competing interests

David Moore is an Associate Editor of BMC Systematic Reviews. None of the authors have any conflicts of interest to declare.

## Authors’ contributions

CD and AT have contributed towards the clinical effectiveness sections of the protocol. SJ and DO have contributed to the cost-effectiveness part of the protocol. SB developed the search strategy. JD and DM have contributed to all aspects of the protocol. All authors have read and approved the final manuscript.
